# Date Palm Tree (*Phoenix dactylifera* L.): Natural Products and Therapeutic Options

**DOI:** 10.3389/fpls.2017.00845

**Published:** 2017-05-23

**Authors:** Reem A. Al-Alawi, Jawhara H. Al-Mashiqri, Jawaher S. M. Al-Nadabi, Badria I. Al-Shihi, Younis Baqi

**Affiliations:** Department of Chemistry, Faculty of Science, Sultan Qaboos UniversityMuscat, Oman

**Keywords:** antioxidant, Arecaceae, cancer, date fruit, palm tree, *Phoenix dactylifera* L.

## Abstract

Many plants, including some of the commonly consumed herbs and spices in our daily food, can be safely and effectively used to prevent and/or treat some health concerns. For example, caffeine the active ingredient found in coffee beans (*Coffea*), shows biological activity in the treatment of the central nervous system (CNS) disorders, indole-3-carbinol, and 3,3′-diindolylmethane are both broccoli (*Brassica oleracea*) derived phytochemicals with potential anti-cancer activity, and resveratrol, isolated from grape (*Vitis vinifera*), is reported to extend lifespan and provide cardio-neuro-protective, anti-diabetic, and anti-cancer effects. Date palm fruits possess high nutritional and therapeutic value with significant antioxidant, antibacterial, antifungal, and anti-proliferative properties. This review focuses on the date fruit extracts and their benefits in individual health promoting conditions and highlights their applications as useful to the pharmaceutical and nutraceutical industries in the development of natural compound-based industrial products.

## Introduction

Date palm tree (*Phoenix dactylifera* L.) is considered as one of the oldest and main staple and ancient crops in Southwest Asia and North Africa. Besides, dates can be grown in Australia, Mexico, South America, southern Africa, and the United States, especially in southern California, Arizona, and Texas (Chao and Krueger, 2007; Al-Harrasi et al., 2014; Hazzouri et al., 2015). Date palm tree belongs to *Arecaceae* family (Angiosperms, monocotyledon) consisting of about 200 genera and more than 2,500 species. *Phoenix* (Coryphoideae phoeniceae) is one of the genera with approximately 14 species (Table 1), which are native to the tropical or subtropical regions of southern Asia or Africa, including *Phoenix dactylifera* L (Siddiq et al., 2013; Eoin, 2016). The name of the species *dactylifera* means “finger-bearing” which refers to the fruit clusters produced by this plant. *Dactylifera* is a grouping of the Greek word *dactylus*, means “finger,” and the Latin word *ferous*, mean “bearing” (Ashraf and Hamidi-Esfahani, 2011). Very recently the whole genome of date palm tree was re-sequenced yields insights into diversification of a fruit tree crop (Hazzouri et al., 2015).

**Table 1:** **Species of the genus ***Phoenix*** along with their common local name and geographical distribution**.

**Species**	**Local name**	**Geographical distribution**
*Phoenix acaulis*	Stemless date palm	Bhutan, Nepal, Northern India
*Phoenix andamanensis*	Andaman Island date palm	Myanmar
*Phoenix atlantica*	Cape Verde Island	Cape Verde Islands
*Phoenix caespitosa*	Date palm	Djibouti, Oman, Saudi Arabia, Somalia, Yemen
*Phoenix canariensis*	Canary Island date palm	Australia, Bermuda, Canary Islands, Italy, Spain
*Phoenix dactylifera L*.	Date palm	Arabian Peninsula, Australia, California, China, El Salvador, Fiji, Iran, India, Mauritius, northern and western Africa, Pakistan, Spain
*Phoenix loureiroi*	Mountain date palm	China, Himalayas, India, Indochina, Philippines
*Phoenix paludosa*	Mangrove date palm	Andaman, India, Indochina, Sumatra
*Phoenix pusilla*	Ceylon date palm	India, Sri Lanka
*Phoenix reclinata*	Senegal date palm	Africa, Arabian Peninsula, Comoros, Madagascar
*Phoenix roebelenii*	Pygmy date palm	China (Yunnan) to North Indo-China
*Phoenix rupicola*	Cliff date palm	Andaman Islands, Bhutan, India
*Phoenix sylvestris*	Indian date palm	Indian Subcontinent, Myanma, southern China
*Phoenix theophrasti*	Cretan date palm	Greek Islands, Turkey

**Figure 1:**
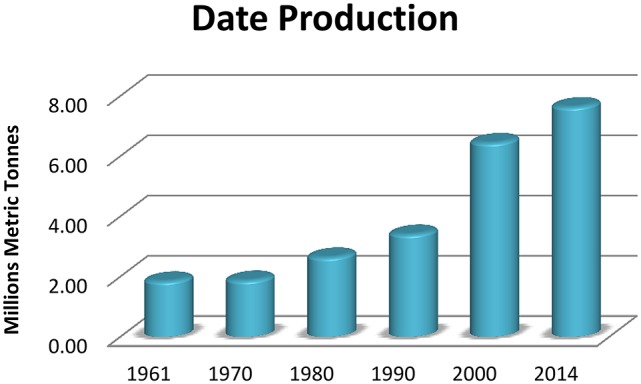
**Date production by the top twenty date fruits producing countries (in metric tons)**.

Flowers of date palm tree are small and yellow colored attached directly to spikelets which develop as fruits called date palm fruits (El Modafar and El Boustani, 2001; Biglari et al., 2007). The world geographical distribution of the genus “*phoenix*” is described in Table 1. Economically and due to the fast growing demand, the production of the dates has been increased over the years. Taking in consideration the top 20 date producing countries, the production of dates was about 3.5 million metric tons in 1990, and 10 years later (in 2000) the world production of the top 20 countries increased to reach almost the double (ca. 6.5 million metric tons), while the latest statistical calculation available, for 2014, is exceeding the seven million and half metric tons (Figure 1) (FAO, http://www.fao.org).

**Figure 2:**
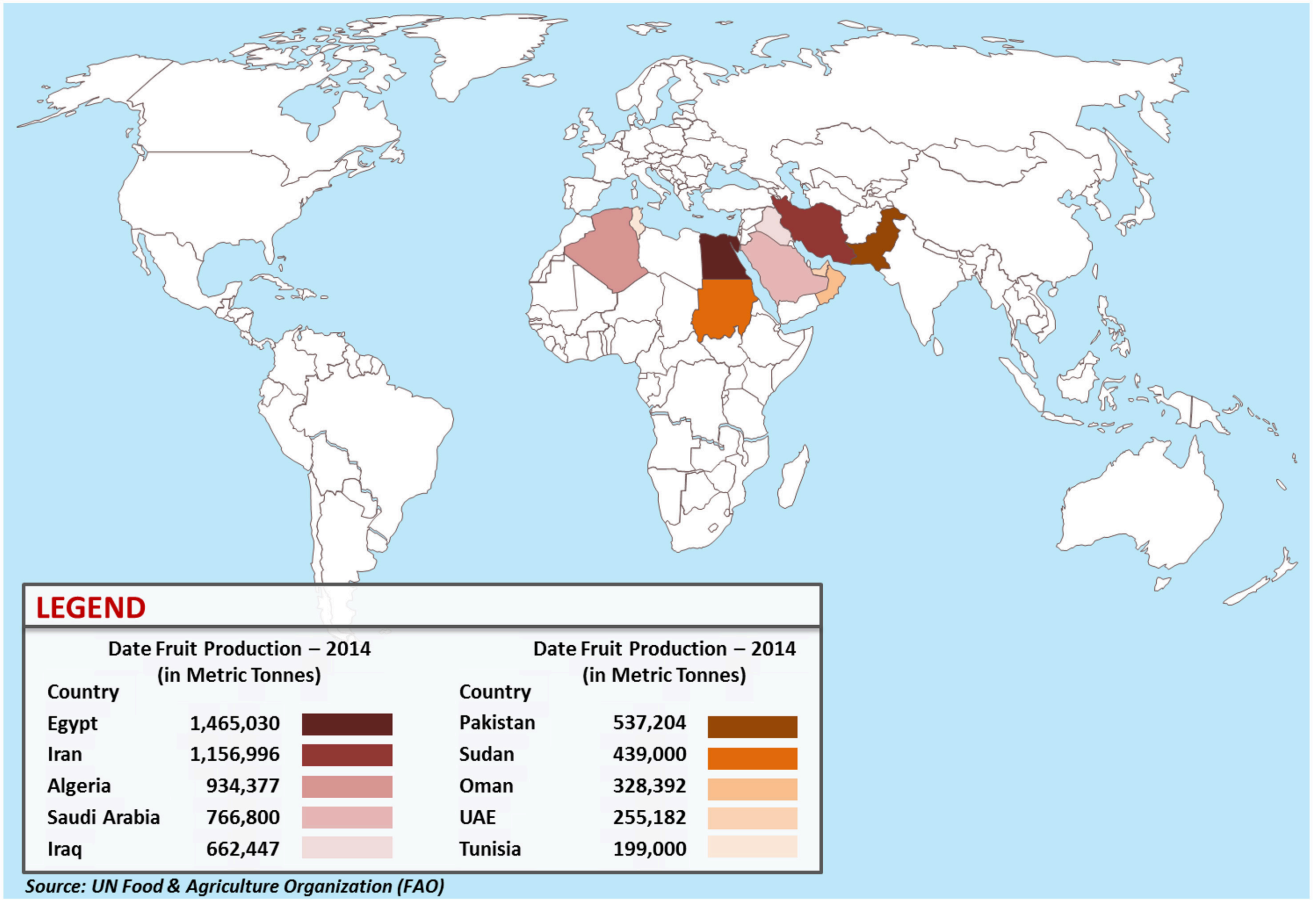
**World map of the top ten date fruit producing countries in 2014**.

Date palm fruits are berry containing a single seed enclosed by fibrous parchment like endocarp, fleshy mesocarp and the fruit skin (pericarp). Different region give different dates which vary in shape, size, and weight. Also, they can vary in their organoleptic, physical and chemical characteristics (Al-Qarawi et al., 2004; Barghini et al., 2007). It is oblong in shape though certain verities might reach near spherical shape. Date palm tree starts fruiting at an average age of 5 years with an average production of 400–600 kg/tree/year and continues to produce for up to 60 years. Egypt, Iran, Algeria, Saudi Arabia, Iraq, Pakistan, Sudan, Oman, UAE, and Tunisia are the top ten date producing countries (Figure 2). Over 100 million date palm trees scatter on ca. 1.3 million hectare worldwide (FAO, http://www.fao.org). The biggest contributed area is the Asian continent including Middle Eastern countries (833,351 hectare), followed by Africa with 416,695 out of which 392, 200 hectare in North Africa alone (Al-Shahib and Marshall, 2013).

**Figure 3:**
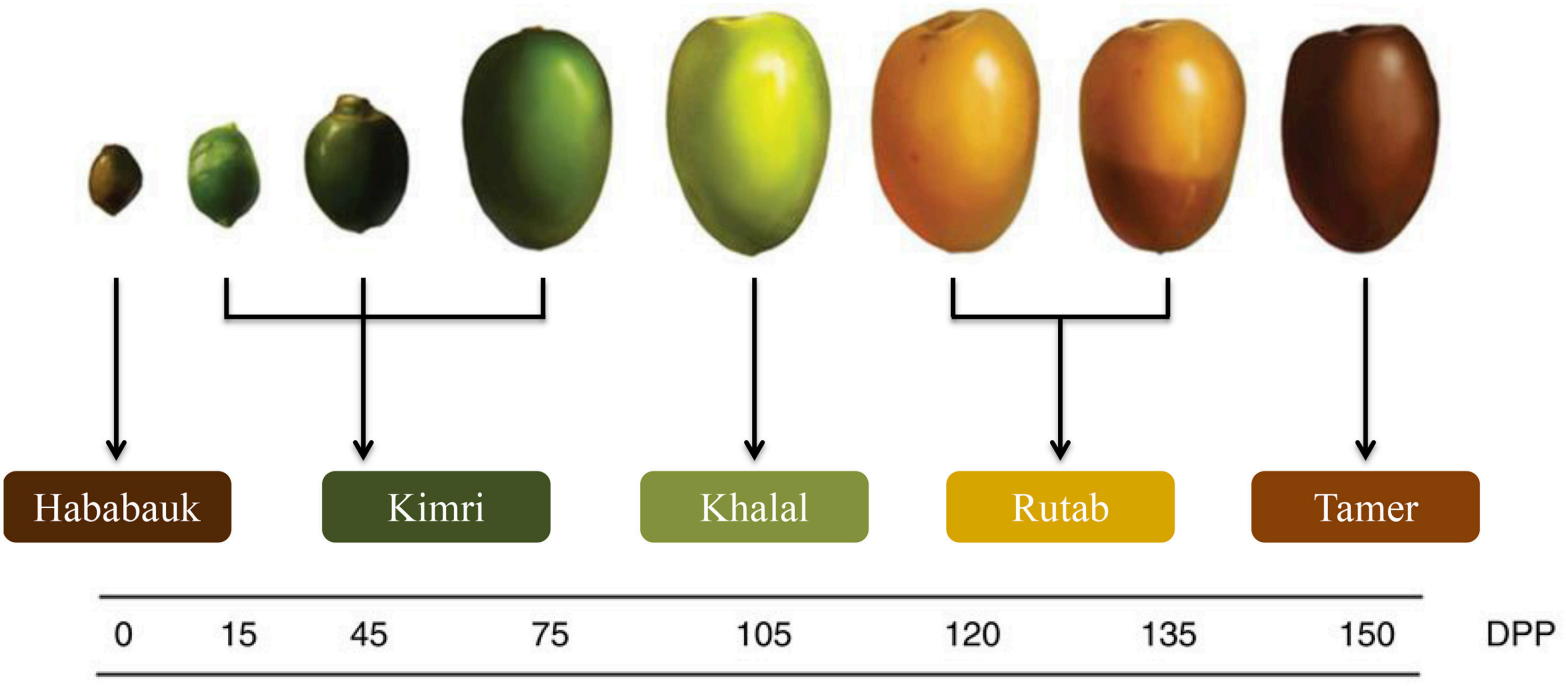
**The five growth stages of a date fruit by days post pollination (DPP) (Al-Mssallem et al., **2013**)**.

The five stages of pre-maturation, maturation and ripening of date are Hababauk, Kimri, Khalal, Rutab, and Tamer (Figure 3; Al-Mssallem et al., 2013). Depending on the maturity and ripeness stages during growth and development of the date, different external and internal changes are observed with color, sweetness, texture and chemical composition (Al-Mssallem et al., 2013; Al-Shahib and Marshall, 2013). Date contains many nutrients such as: carbohydrates, proteins, fat, minerals and vitamins (Al-Qarawi et al., 2004; Barghini et al., 2007).

The most important quality attributes to grade dates are color, flavor (sugar level), moisture (26–30%) and absence of defects such as insect, damage, cracks and surface damage. Date fruit is good source of high nutritional value food. Indeed it is rich in carbohydrates, dietary fibers, proteins, minerals and vitamin B complex, such as thiamine (B_1_), riboflavin (B_2_), niacin (B_3_), pantothenic (B_5_), pyridoxine (B_6_), and folate (B_9_) (Chao and Krueger, 2007; Al-Harrasi et al., 2014; Siddiq et al., 2013; Eoin, 2016). In more details, carbohydrates forms 70% of date fruit and are mostly fructose and glucose in equal ratio while date proteins are rich in amino acids that contain acidic side chain but poor in methionine and cysteine, which their side chain composed of sulfur. Minerals in date fruits are calcium, iron, magnesium, selenium, copper, phosphorus, potassium, zinc, sulfur, cobalt, fluorine, manganese, and boron (Chao and Krueger, 2007; Al-Harrasi et al., 2014; Siddiq et al., 2013; Eoin, 2016). The edible part of the date palm tree has been recognized to possess many medicinal properties when consumed alone or in mixture with other medicinal herbs (Tiwari et al., 2013). In recent years, a huge interest in the abundant health promoting properties of date fruits had led to many pharmacological studies (*in-vitro* and *in-vivo*) as well as the identification and quantification of different classes of phytochemicals (Chao and Krueger, 2007). The date fruits are highly nourishing and may have numerous potential health benefits. The protective effects of fruits against chronic diseases are ascribed to bioactive non-nutrients called phytochemicals. Phytochemicals have gained increased interest among several investigators, including clinicians due to their antioxidant activity, cholesterol-lowering properties, and other potential health benefits such as chemoprevention of cancer, prevention of diabetes and cardiovascular diseases (Chao and Krueger, 2007; Tiwari et al., 2013; Al-Harrasi et al., 2014).

## Phytochemicals in date palm fruits

Phytochemicals are plant-derived chemicals which may give health benefits when taken as a medicine drug or as a part of daily diet. They are classified into two main categories: primary metabolites, which occur in all cells and play an essential role in the reproduction and metabolism of those cells, for example nucleic acids, the common amino acids and carbohydrates (sugars); secondary metabolites such as terpenes (a group of lipids), phenolics (derived from carbohydrates), alkaloids (derived from amino acids) which are characteristic of a limited range of species and have a biological effect on other organism (Thatoi and Patra, 2011; Dias et al., 2012). Many of biologically active constituents of medicinal, commercial and poisonous plants are classified as secondary metabolites. Date fruit is rich in phytochemicals such as carotenoids, polyphenols (e.g., phenolic acids, isoflavons, lignans, and flavonoids), tannins, and sterols (Martín-Sánchez et al., 2014). The concentration and composition of these constituents are widely varied depending on several parameters, including date variety, stage of fruit picking, storage, postharvest processing, the geographical origin of the dates and soil conditions (Al-Laith, 2009; Amorós et al., 2009; Al-Turki et al., 2010). Several researchers have reported that the chemical constituents and functional composition of date fruits are dramatically changed during date maturing period with increasing in levels of reducing sugars, while fiber, mineral, and vitamin levels decreasing steadily (Kikuchi and Miki, 1978; Al-Farsi et al., 2007; Al-Turki et al., 2010).

### Carotenoids

**Figure 4:**
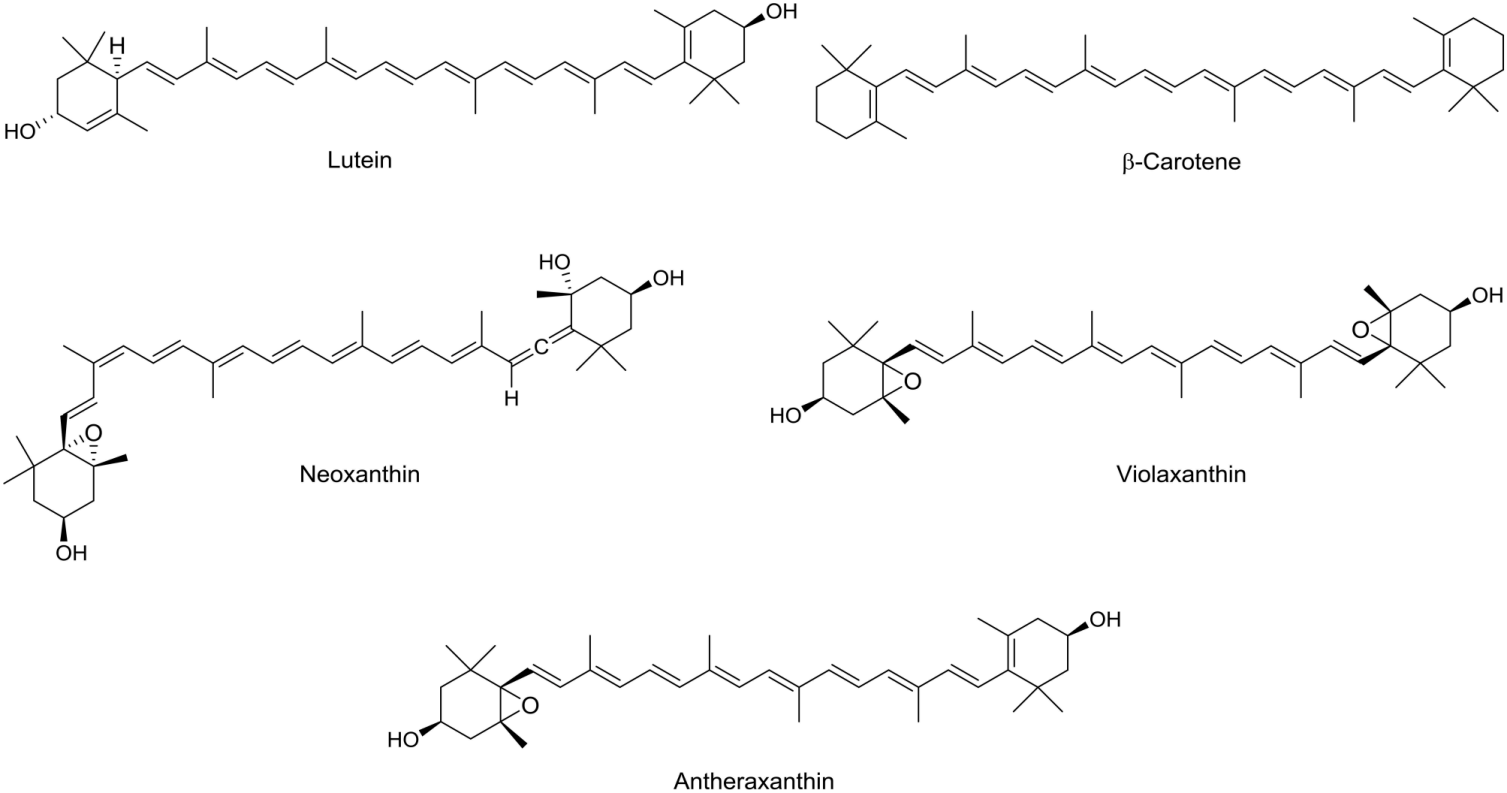
**Chemical structures of carotenoids identified in date fruits**.

Carotenoids considered as a major class of phytochemicals occur in the lipid fractions of date fruit. They are precursors of vitamin A, which plays a central role in vision, and protects the cell from deleterious effects of free radicals by acting as antioxidants (Julia et al., 2015). Carotenoid classification is depending on the presence or absence of oxygen in the molecule, they can be divided into two main subclasses: xanthophylls (contain oxygen atom) and carotenes (lack oxygen atom). Boudries et al. studied the carotenoid composition for three different varieties at three edible maturation stages (Khalal, Rutab, and Tamr) and they found that dates contain lutein and β-carotene as major carotenoids (Boudries et al., 2007). Neoxanthin, violaxanthin, and antheraxanthin were identified in date fruits in lesser amount (Figure 4). Al Farsi et al. analyzed the total carotenoids for three date fruit varieties (Fard, Khasab, and Khalas) and they found that Khalas has the highest amount of carotenoids as expected as this variety has a yellow color. They also reported destruction of total carotenoids after sun drying of date fruit ranged between 4 and 30% (Al-Farsi et al., 2005a). Dried date fruit is a moderate source of carotenoid (0.97 mg/100 g) compared to other dried fruits, e.g., figs and apricot: 0.032 mg/100 g and: 2.20 mg/100 g respectively (Martín-Sánchez et al., 2014).

### Phytosterols and phytoestrogens

**Figure 5:**
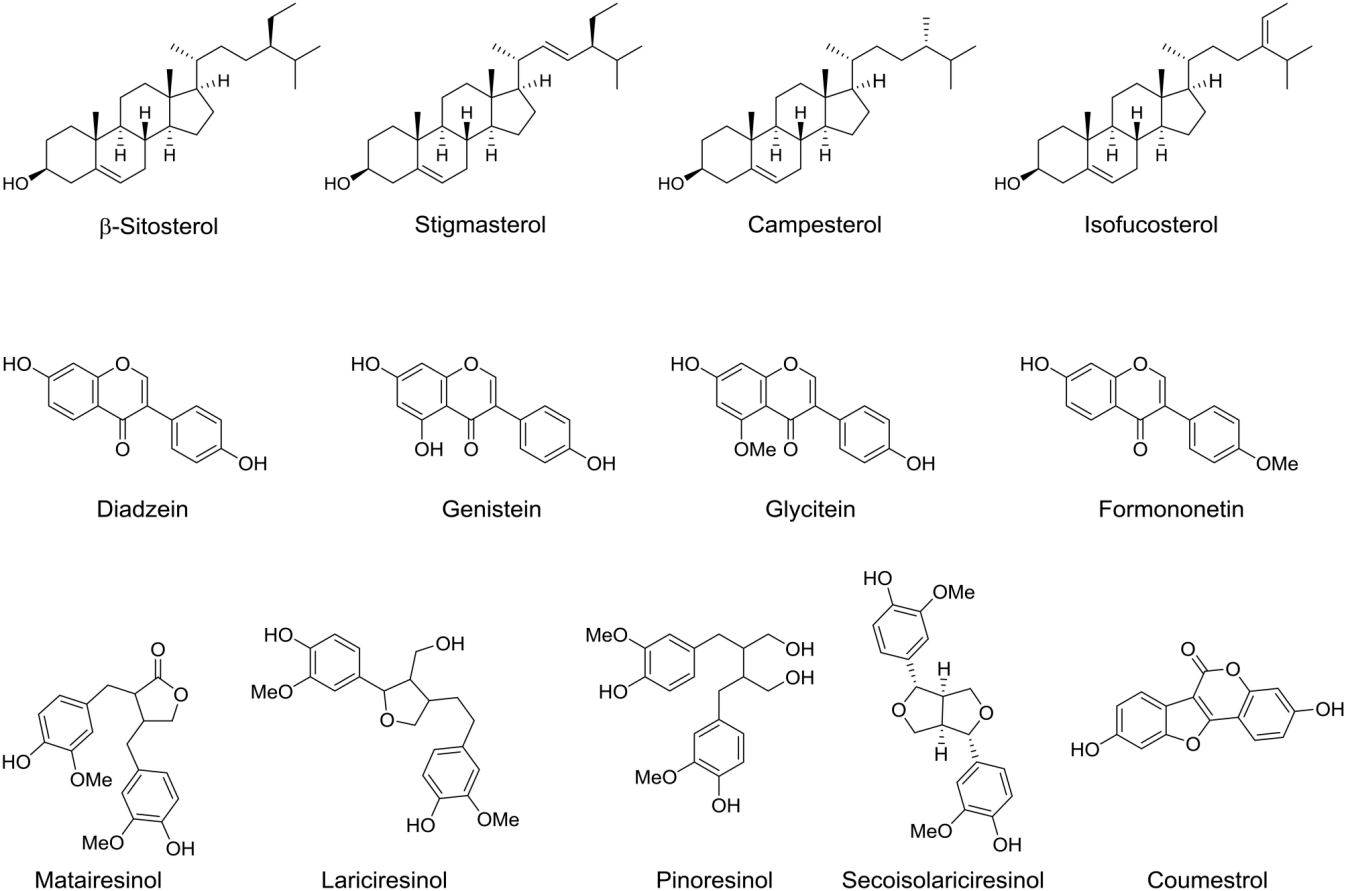
**Chemical structures of phytoestrols and phytoestrogens isolated/identified in the fruit of date palm tree**.

Phytosterols are another major phytochemicals that found in the lipid soluble fraction of the date fruit. These compounds are exclusively occurring in plants with chemical structure similar to that of cholesterol (Al-Laith, 2009). Approximately 200 phytosterols exist in nature and many of them are found in vegetables and fruits (Amorós et al., 2009). Date fruit at tamer stage contains several phytosterols. Back in 1978, cystalline plant sterol mixture was first isolated from the edible part of date fruit and has been identified to include β-sitosterol, stigmasterol, campesterol, and isofucosterol (Figure 5; Kikuchi and Miki, 1978). However, the difference in the phytosterol composition among the date fruit varieties and its ripening stages is still unclear and therefore make it an important line for advance research. Moreover phytoestrogens are natural compounds that can bind estrogen receptors and exert diverse estrogenic or antiestrogenic effects (Al-Turki et al., 2010). Thompson *et al*. studied the phytoestrogens content in date fruit and they identified several phytoestrogens including formononetin, daidzein, genistein, glycitein, matairesinol, lariciresinol, pinoresinol, secoisolariciresinol, and coumestrol (Figure 5; Thompson et al., 2006).

### Phenolic acids

**Figure 6:**
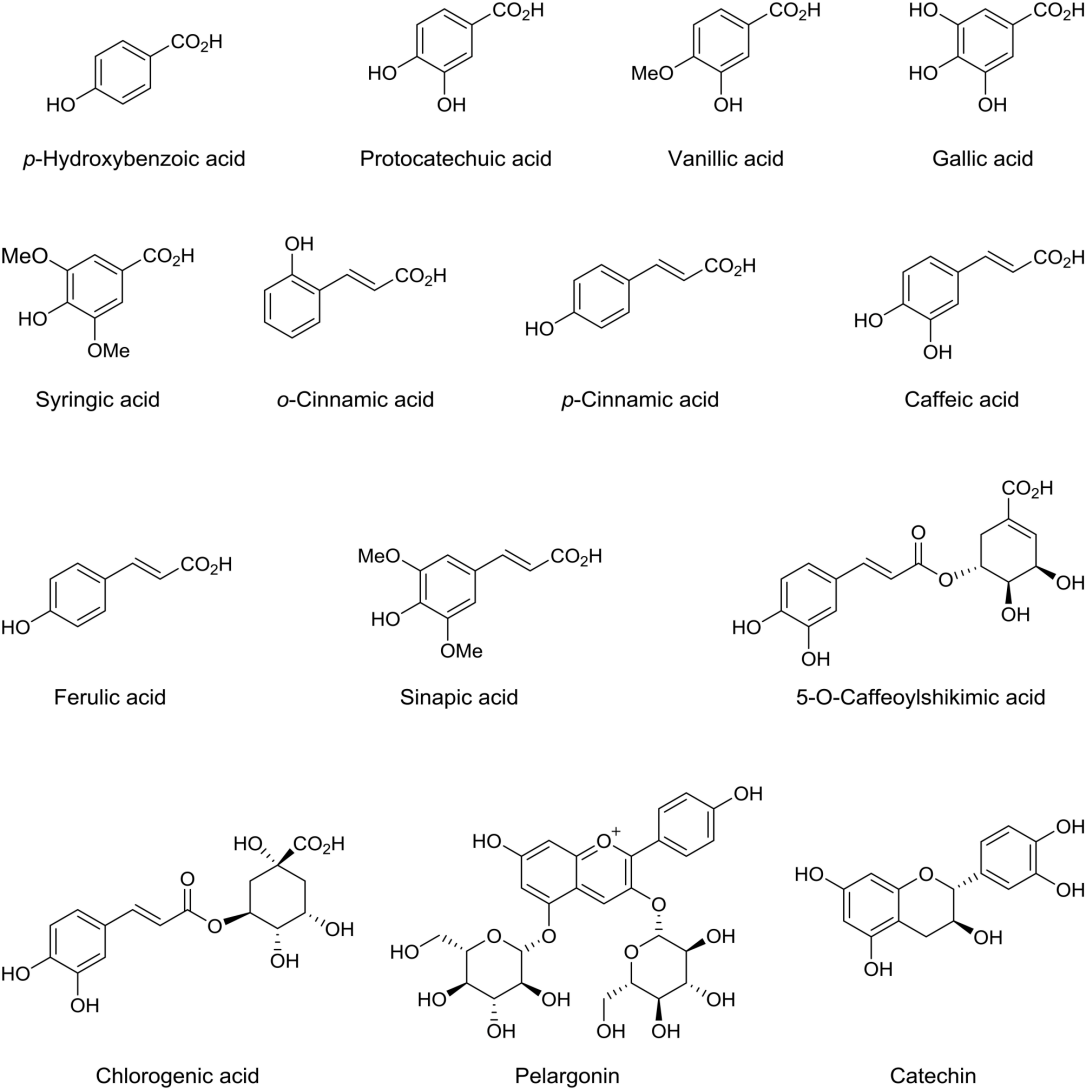
**Chemical structures of phenolic acid compounds identified in date fruits**.

Phenolic acids considered as one of the main aromatic secondary plant metabolites, containing hydroxyl function located on aromatic benzene ring with one or more carboxylic acid groups. Phenolic acids can be divided into two main classes: benzoic acid derivatives of which contain seven carbon atoms and cinnamic acid derivatives of which contain nine carbon atoms. They considered as effective antioxidant because they act as free radical captor or scavenger. Several research groups reported that dates are rich in phenolic acids (Saleh et al., 2011; Benmeddour et al., 2013; El Sohaimy et al., 2015). Al Farsi et al. studied three different Omani date fruits (Fard, Khasab, and Khalas) and found to contain the following benzoic acid derivatives; *p*-hydroxybenzoic acid, protocatechuic acid, vanillic acid, gallic acid and syringic acid, while the cinnamic acid derivatives were; *o*-coumaric acid, *p*-coumaric acid, caffeic acid, and ferulic acid (Figure 6; Al-Farsi et al., 2005a). In another study of seven different varieties of date fruits cultivated in Algeria, Mansouri et al. identified the main phenolic acids including *p*-coumaric acid, ferulic acid and sinapic acid. Moreover, three different isomers of 5-o-caffeoyl shikimic acid were identified, in addition xanthoxylin acid, hydrocaffeic acid, and coumaroylquinic acid were reported (Figure 6; Mansouri et al., 2005). Karasawa et al. identified protocatechuic acid, syringic acid, caffeic acid, ferulic acid, and chlorogenic acid in the date extract using UPLC by comparing the retention time and UV spectrum of the peaks of these compounds with those of standard phenolic acids (Figure 6; Karasawa et al., 2011). The major phenolic acids in Saudi date fruit varieties were gallic acid, *p*-coumaric acid, and ferulic acid derivatives (Hamad et al., 2015). Recently, Borochov-Neori et al. studied the phenolic composition of two date varieties, Amari and Hallawi, at Tamr stage. The phenolic fractions have been analyzed by RP-HPLC using system software based on UV/Vis absorbance spectra and the retention times of the chromatogram peaks were compared with library of authentic related standard compounds. The quantity of each individual phenolic compounds were calculated from the corresponding area under the curve of the chromatogram peak with the help of the software in triplicate runs, and compared with authentic standards of caffeic acid, coumaric acid, ferulic acid, and salicylic acid and kaempferol-3-glucoside. They detected five and seven phenolic acids constituent in Hallawi and Amari, respectively. The major component of the phenolic fraction was ferulic acid and a trace amount of coumaric acid for both varieties. Amari found to contain mostly caffeic acid derivatives, while salicylic acid was the most abundant phenolic acid in Hallawi (Borochov-Neori et al., 2015). Protocatechuic acid, vanillic acid, gallic acid, syringic acid and *p*-coumaric acid were detected in three different Tunisian date fruit varieties (Figure 6; Mrabet et al., 2016). Lemine et al. found that the amount of phenolic acids in Khalal stage of date is significantly higher than in the fully mature Tamer stage, 0.729 g/100 g, and 0.559 g/100 g (wt/wt), respectively (Lemine et al., 2014).

Moreover, Awad et al. reported that the quantity of phenolic compounds decreased, with a 25% loss through the ripening stages from the Khalal to the Tamar in date fruits cultivar from Tunisia (Awad et al., 2011). However, Al-Najada and Mohamed studied the change of total phenolic contents of Khalas and Shishi date fruits during storage at 4°C and they reported that the phenolic contents significantly increased after 6 months, while after 12 months of storage the phenolic contents increase up to ca. double the amount (Al-Najada and Mohamed, 2014).

### Flavonoids

**Figure 7:**
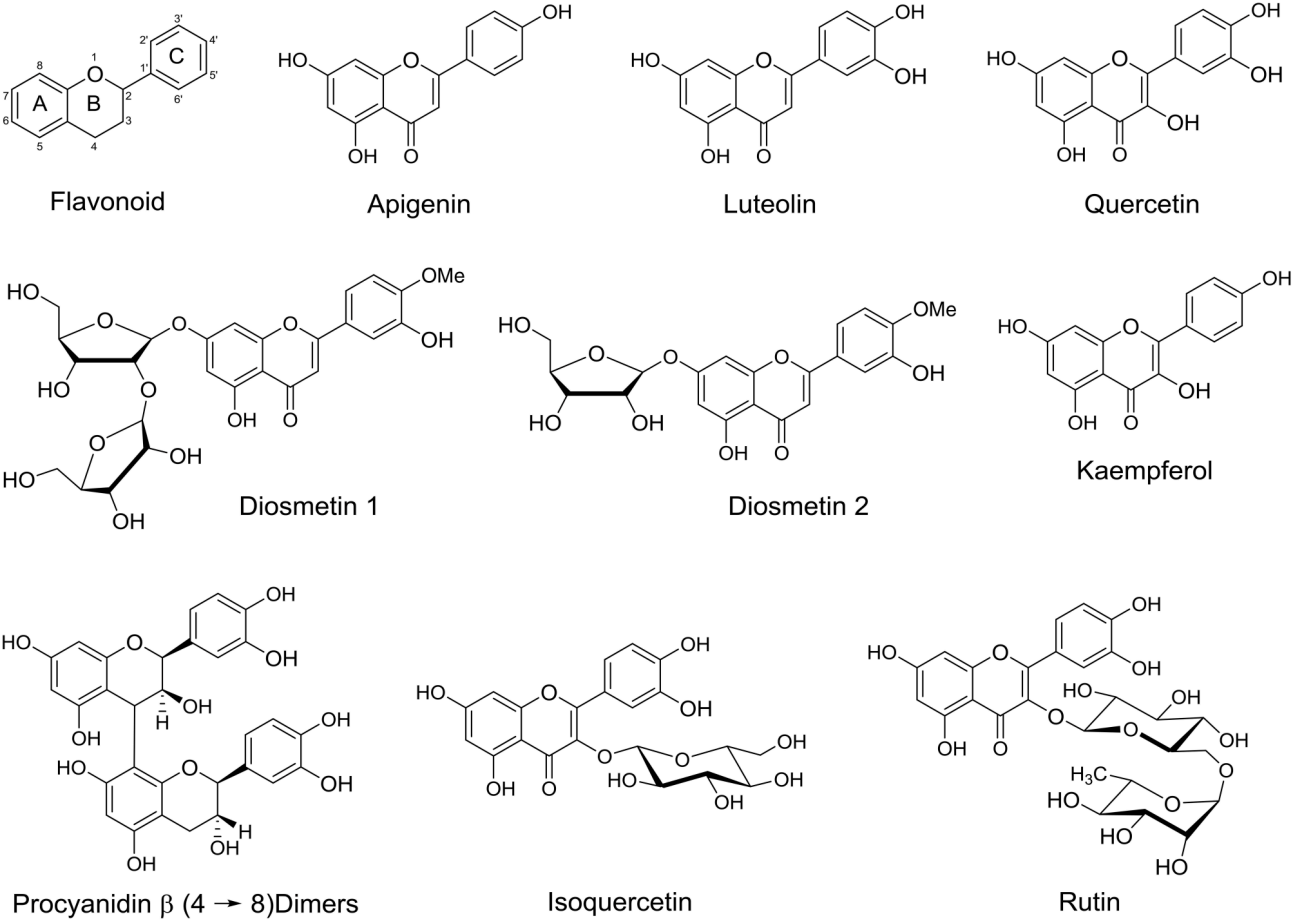
**Basic flavonoid skeleton and some flavonoids identified in date fruits**.

Flavonoids are large family of polyphenolic plant derived secondary metabolites, comprise of 15 carbons skeleton containing two aromatic benzene rings A and C chemically bound via a heterocyclic pyrane ring C and this skeleton is often substituted with multiple substitution patterns (Figure 7). Flavonoids are classified into several subgroups, including flavones, flavonols, flavanones, flavanonol, isoflavones, isoflavonone, flavan-3-ols, and anthocyanidins. Flavonoids are found in a variety of fruits and vegetables with notable health benefits as antioxidant and anti-inflammatory (Moss and Ramji, 2016). Hong *et al*. studied the flavonoid glycoside and procyanidin compositions of date fruit, variety Deglet Noor, harvested at Khalal stage of maturity by liquid chromatography-electrospray ionization/tandem mass spectrometry (LCESI/MS/MS) and found that it contain 13 flavonoid glycosides of apigenin, luteolin, and quercetin, 19 in isomeric forms (Figure 7), in addition they reported flavonoid sulfates (Hong et al., 2006). Chaira et al. reported that Korkobbi variety has the highest level of flavonoids among 10 Tunisian date varieties, as a consequence, it shows the highest antiradical efficiency of this cultivar (Chaira et al., 2009). In Oman, the total flavonoid content has been investigated for three major date varieties; Fardh, Khasab, and Khalas at two edible maturation stages; Rutab and Tamr (Singh et al., 2012). Michael et al. found a new diosmetin glycosides; diosmetin 7-O-β-L-arabinofuranosy (1→2)-β-D-apiofuranoside (Diosmetin 1) and diosmetin 7-O-β-D-apiofuranoside (Diosmetin 2), which was isolated from acetone extract of date fruits (Figure 7; Michael et al., 2013).

In another study on 11 different Saudi date fruit varieties, apigenin, luteolin, quercetin, isoquercetrin, and rutin were identified (Hamad et al., 2015). Kaempferol, a natural flavonol, derivatives were identified in Amari and Hallawi date fruits, where Amari contained five flavonol in a significant amount and Hallawi contained one major flavonol (Borochov-Neori et al., 2015). Lemine et al. reported that the total content of flavonoid decrease significantly from Khalal stage to Tamer stage for the seven different date fruits varieties that they examined (Lemine et al., 2014). Another study showed that the flavonoid content reduced during maturity stages from Khalal to Tamer stage for four different studied Tunisian date fruit varieties (Amira et al., 2012). Al Farsi et al. measured the content of anthocyanins in fresh and dried date fruits and these were identified only in fresh dates (Al-Farsi et al., 2005a), this might be due to the destruction after expose it to the sun. It was reported that enzymatic and non-enzymatic browning reactions can cause the destruction of anthocyanins during drying and storage period (Wrolstad, 2004). While pelargonin, an anthocyanin was identified in date fruits (Figure 6; Karasawa et al., 2011). Moreover, the total flavanol, including catechin in the edible part found to be more than that in the date pits (Hammouda et al., 2013).

## Natural products

**Figure 8:**
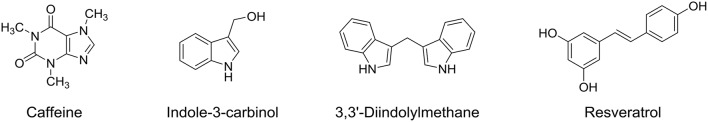
**Selected plant derived natural products with paramount health promoting activity**.

Natural products are organic derived compounds produces *in-vivo* by primary or secondary metabolism, which usually carried out by biocatalytic pathway involving a biocatalyst (enzyme). Since natural products are naturally biosynthesized, these found to interact with very crucial biopolymer molecules in the living cells, such as proteins (enzymes, receptors), DNA and RNA, which these are the same targets for drugs (Mrabet et al., 2016). For example, caffeine the active ingredient found in coffee beans (*Coffea*), shows biological activity in the treatment of the central nervous system (CNS) disorders (Silva et al., 2013; Kaster et al., 2015), indole-3-carbinol and 3,3′-diindolylmethane are both broccoli (*Brassica oleracea*) derived phytochemicals with potential anti-cancer activity (Wang et al., 2012; Mayne et al., 2016), and resveratrol, isolated from grape (*Vitis vinifera*), is reported to extend lifespan and provide anti-diabetic, cardio-neuro-protective, and anti-cancer effects (Figure 8) (Sajish and Schimme, 2015; Dance, 2016).

It has been estimated, that 62% of all modern drugs are of natural products origin, of which (14%) are mimic of natural product or containing natural product pharmacophore. The rest 38% of current drugs are either purely synthesized (27%) or synthesized to mimic a natural product (11%) (Newman and Cragg, 2016). Thereby documenting that natural product compounds have been a successful drug source for the pharmaceutical industry. Moreover, world-health organization reported that ca. 80% of the world's populations depend on conventional medicine for their primary health care (Awad et al., 2011). Natural products extracted from the fruits of date palm tree has been shown to possess many health promoting properties such as anti-inflammatory, anti-fungal, anti-oxidant and anti-tumor effects (Al-Qarawi et al., 2004; Vayalil, 2012; Rahmani et al., 2014).

## Therapeutic options

Antioxidants have received great attention because they act as free radicals scavenger related to various diseases including cancer (Gorrini et al., 2013), heart diseases (Moss and Ramji, 2016), Alzheimer's (Frost et al., 2014), and Parkinson's disease (Kim et al., 2015). Oxidative stress happens when the production of reactive oxygen species (ROS) is greater than the ability of the body to detoxify the reactive intermediates. The body naturally produces antioxidants such as, superoxide dismutase (SOD), catalase, glutathione peroxidase (GSHPx) to protect itself against free radicals. The antioxidants neutralize the free radicals, so rendering them harmless to other cells (Nathan and Cunningham-Bussel, 2013). However these endogenous antioxidants are not enough to neutralize all of the free radicals generated in the body.

A daily diet intake rich in vegetables and fruits has been shown to reduce oxidative damage of DNA bases in humans (Kotepui, 2016), protective for heart disease (Harasym and Oledzki, 2014) and protect against lipid peroxidation (Basu et al., 2014). The antioxidant activity of phenolic compounds is found to be comparable to the standard antioxidants, such as vitamin C, vitamin E, and β-carotene (Rautiainen et al., 2016). Earlier investigation reported that date fruits have the highest concentration of total polyphenols among the dried fruits due to the greater exposure to sunlight and extreme temperature for date fruits compared to other fruits (Vinson et al., 2005). However, their composition can be vary from cultivar to cultivar depending on soil conditions and agronomic practice, for example, the nutritional quality of date fruits alters among varieties grown in Algeria (Saleh et al., 2011) and Oman (Al-Farsi et al., 2005b; Chaira et al., 2009), Bahrain (Allaith, 2008), and Sudan (Mohamed et al., 2014). Guo et al. studied the antioxidant activities of 28 fruits commonly consumed in China and they found that date fruits possess the second highest antioxidant activity after Hawthorn (Guo et al., 2003). In another study, Saafi et al. investigated the effect of aqueous date fruit extract (Deglet Noor variety) on the protection against oxidative damage as well as hepatotoxicity induced by subchronic exposure to dimethoate on rat liver and the data showed that the extract repaired the damage of the liver (Saafi et al., 2011). In different study, Mansouri et al. examined the immunomodulatory effects of a hot water extract from date at tamer stage, prune and fig fruit in mice (Mansouri et al., 2005). They found that date fruit extract promote the cellular system more than prune and fig extract. Moreover, it has been reported that diosmetin glycosides (diosmetin 1 and 2, Figure 5) can increase the insulin excretion and stimulate the enzyme glycogen synthase, which maintain homeostasis of blood glucose levels *in-vivo* using alloxan diabetic male rats (Singh et al., 2012). In addition, the treatment of alloxan diabetic male rats by these two compounds showed a highly increase in serum testosterone level accompanied with a significant decrease in total and prostatic acid phosphate activities (Singh et al., 2012). El Sohaimy et al. measure the antimicrobial activity of some Egyptian date fruit against five pathogenic bacterial strains and they found that the water and ethanol extracts has a strong antibacterial activity (El Sohaimy et al., 2015). Al-Yahya et al. studied the cardioprotective effect of lyophilized aqueous date fruit extract (Ajwa variety) *ex-vivo* and *in-vivo* and they found that it enhance the cardiomyoblast cell proliferation by up to 40%, prevented the consumption of endogenous antioxidants and inhibited lipid peroxidation (Al-Yahya et al., 2015). Garba and Galadima investigated the antimicrobial potential of date fruit extract on the bacteria, *salmonella* spp. and *shigella* spp., which cause diarrhea. The extracts from water, methanol and petroleum ether showed a significant activity as anti-diarrhea (Garba and Galadima, 2012). Borochov-Neori *et al*. investigated the antioxidant and antiatherogenic properties of date fruit (Amari and Hallawi varieties) at Tamr stage extract, prepared by acetone/water (7:3, v/v) solution containing 0.5% acetic acid. Several methods have been used to measure the antioxidant activities of these varieties and were reported to possess antioxidant activity comparable to vitamin C (Borochov-Neori et al., 2015). Moreover, they studied the effect of the isolated phenolic acid and flavonol fractions from date fruits in the extent of cholesterol efflux and they found that the flavonol fractions enhanced cholesterol removal from macrophages. This positive antioxidant effect might protect the cell membrane from being oxidized by the effect free radicals generated both extra- and intracellularly (Borochov-Neori et al., 2015). Lemine et al. studied the anti-oxidant activity of seven different Mauritanian date fruits varieties at two edible stages (Khalal and Tamr) and they found that Khalal has higher antioxidant compare to Tamr stage (Lemine et al., 2014). While Al Farsi et al. reported the antioxidant activity of three date fruit varieties grown in Oman, namely; Fard, Khasab, and Khalas. The results show that Khalas to possess higher antioxidant activity (Al-Farsi et al., 2005a).

Date fruit may have a potential health benefits against many types of cancer as it suggested by experimental evidence and the phytochemical composition. Al-Sayyed et al. reported the potential cancer preventive effects of date fruit. They found that increase consumption of dried date fruit reduced significantly the incidence rate of mammary cancer, palpable tumor multiplicity, tumor size and weight compared to the positive control group (Al-Sayyed et al., 2014). Eid et al. studied the whole date fruit extract and its polyphenol-rich extract, both extracts were prepared from methanol/water (4:1, v/v) containing 10% of 1 molar sodium fluoride (NaF) solution, found to inhibit Caco-2 cell growth, indicating that both were capable of probably acting as anti-proliferative agents *in-vitro*. According to their results, consumption of date fruits may promote health condition of the colon by increasing the growth of beneficial bacterial and inhibiting the proliferation of colon cancer cells (Eid et al., 2014). In another study, Zangiabadi et al. studied the effect of aqueous date extract on diabetic polyneuropathy in streptozotocin induced diabetic rats and they reported that date extract was able to prevent the diabetic aggravation and in enhancing pathological parameters of diabetic neuropathy (Zangiabadi et al., 2011). Pujari et al. reported that date extract, prepared using methanol/water (4:1, v/v), gives significant neuroprotection against cerebral ischemia induced by bilateral common carotid artery occlusion (Pujari et al., 2011). Souli et al. suggested that aqueous date fruit extract accelerate the gastrointestinal transit activity and reduces the risk of constipation (Souli et al., 2014). In a very recent study, Belmir et al. investigated the antifungal activity of aqueous date fruit (Ajwa variety) extract at Tamr stage with the amphotericin B, an antifungal drug. They reported that therapeutic index of amphotericin B increased significantly with the extract and the cytotoxicity induced by amphotericin B test showed that the aqueous date extract prevents cytotoxicity of red blood cells (Belmir et al., 2016). Kchaou et al. examined the cytotoxic property of Tunisian date fruits, using methanol/water (4:1, v/v), and the result showed that the human cells growth has been significantly decrease after treatment with the date extract (Kchaou et al., 2016). In another study the antibacterial properties and antioxidant activity of Saudi date fruits variety Ajwa, Safawi, and Mabroom, as well as Iranian date fruits variety Mariami was investigated. The date fruits were treated with polar extraction solvents (methanol or acetone) at 4°C, while the extracted materials were stored at −20°C. The storage stability of total anthocyanin content (TAC) was also studied and shows that date variety Mariami had the highest TAC content while Mabroom had the lowest TAC. The total amount of extracted phenolic compounds from dates using methanol as solvent found to be a better solvent compared to acetone. It was reported that different cultivars exhibited different antibacterial properties. The methanolic extract of Ajwa date variety was reported to exhibited antibacterial activity among all studied bacteria: *Escherichia coli, Bacillus cereus, Staphylococcus aureus*, and *Serratia marcescens* (Samad et al., 2016). A recent study by Taleb et al. investigated the pharmacological effect of date syrup, which made from the edible part of date fruit, and has been found to be useful in the treatment of several diseases with etiologies involving inflammation and angiogenesis. The authors reported that the polyphenolic compounds occur in date syrup reduce angiogenic responses, e.g., tube formation, cell migration, and matrix metalloproteinase activity in an inflammatory model by exhibiting anti-inflammatory activity mediated by the prostaglandin enzyme cyclooxygenase-2 (COX-2) and vascular endothelial growth factor (VEGF) in endothelial cells. Inflammation was found to be reduced by the administration of the date fruit syrup polyphenolic compounds at 60 and 600 μg/mL, as well as suppressed many stages of angiogenesis, including endothelial matrix metalloproteinase activity, invasion, tube formation and cell migration. Interestingly, date syrup shows no cytotoxicity effect. Moreover, the polyphenolic compounds of date syrup were found to significantly reduce the expression of COX-2 and VEGF induced by tumor necrosis factor-alpha at both protein level and gene expression in comparison to untreated cells (Taleb et al., 2016). Most recently a study by Khan et al. reported the effects of date, Ajwa variety, on cancer therapy; therefore date fruits might be used as useful as an adjunct therapy with conventional chemotherapeutics to achieve a synergistic effect against breast cancer (Khan et al., 2016).

## Perspectives

*In-vitro* and *in-vivo* studies of several pure aqueous and mixed aqueous/organic solvent extracts of the date palm fruits were found to possess many health promoting effects; including oxidative stress activity, free radical scavenging capacity, coronary heart disease prevention, hepatoprotective, anti-inflammatory, and anticancer activities. However, the non-mixed organic solvent extracts of date fruits are currently not sufficiently covered in the literature. Organic solvents extracts of date fruits will minimize the carbohydrate, which is highly water soluble, concentration in the extracted materials. After multi-gram scale organic solvents extract materials, a systematic isolation and identification procedures need to be followed up to isolate the pure single organic compound, which is responsible for the biological activity. The reproduction of the most active ingredient in a chemistry laboratory, then industrially, will help in finding cheaper way to develop new drugs and saving the nature, especially endangered species, by which we can protect the medicines of the wild pharmacy.

## Author contributions

RA, JHA, JSA and BA extracted data from literature and helped in draw the chemical structures of the compounds. YB wrote and finalized the manuscript.

## Copyright statement

The appropriate permission has been obtained from the nature publishing group for the reproducing of Figure 3.

### Conflict of interest statement

The authors declare that the research was conducted in the absence of any commercial or financial relationships that could be construed as a potential conflict of interest. The reviewer KK and handling Editor declared their shared affiliation, and the handling Editor states that the process met the standards of a fair and objective review.
